# Rehabilitation after surgical treatment of peroneal tendon tears and ruptures

**DOI:** 10.1007/s00167-015-3944-6

**Published:** 2016-01-23

**Authors:** Pim A. D. van Dijk, Bart Lubberts, Claire Verheul, Christopher W. DiGiovanni, Gino M. M. J. Kerkhoffs

**Affiliations:** Department of Orthopaedic Surgery, Orthopaedic Research Center Amsterdam, Academic Medical Center, University of Amsterdam, Meibergdreef 9, 1105 AZ Amsterdam, The Netherlands; Academic Center for Evidence Based Sports Medicine (ACES), Amsterdam, The Netherlands; Amsterdam Collaboration on Health and Safety in Sports (ACHSS), Amsterdam, The Netherlands; Foot and Ankle Service, Department of Orthopaedic Surgery, Massachusetts General Hospital, Boston, MA USA; Orthopaedic Manual Therapy and (Sport)Physiotherapy, ManualFysion, Amsterdam, The Netherlands

**Keywords:** Physical therapy, Rehabilitation, Peroneal tendon, Tear, Tendon, Rupture, Tendon healing

## Abstract

**Purpose:**

The purpose of this study was to provide an overview of the available evidence on rehabilitation programmes after operatively treated patients with peroneal tendon tearsand ruptures.

**Methods:**

A systematic review was performed, and PubMed and EMBASE were searched for relevant studies. Information regarding the rehabilitation programme after surgical management of peroneal tendon tears and ruptures was extracted from all included studies.

**Results:**

In total, 49 studies were included. No studies were found with the primary purpose to report on rehabilitation of surgically treated peroneal tendon tears or ruptures. The median duration of the total immobilization period after primary repair was 6.0 weeks (range 0–12), 7.0 weeks (range 3.0–13) after tenodesis, 6.3 weeks (range 3.0–13) after grafting, and 8.0 weeks (range 6.0–11) after end-to-end suturing. Forty one percent of the studies that reported on the start of range of motion exercises initiated range of motion within 4 weeks after surgery. No difference was found in duration of immobilization or start of range of motion between different types of surgical treatment options.

**Conclusion:**

Appropriate directed rehabilitation appears to be an important factor in the clinical success of surgically treated peroneal tendon tears and ruptures. There seems to be a trend towards shorter immobilization time and early range of motion, although there is no consensus in the literature on best practice recommendations for optimizing rehabilitation after surgical repair of peroneal tendon tears or ruptures. It is important to adjust the rehabilitation protocol to every specific patient for an optimal rehabilitation.

*Level of evidence* Systematic Review, Level IV.

## Introduction

Lateral ankle sprains are among the most common acute musculoskeletal injuries [9] and can result in peroneal tendon disorders, particularly peroneus brevis tendon tears. The exact prevalence of these tears in general population remains unknown, but cadaveric studies have shown a prevalence of 11–38 % [32, 57]. Surgical treatment is essential to prevent deterioration of tendon tissue and chronic pain complaints [13, 25, 46, 64]. To our knowledge, there is no consensus in the available literature regarding appropriate after-treatment of surgically treated peroneal tendon tears.

Acute ankle inversion injury is a typical trigger for a peroneal tendon tear. Chronic lateral ankle instability with repetitive sprains, repetitive stress or overuse, peroneal tendon subluxation, or anatomic abnormalities can also provoke tears [17, 25, 51, 55, 58, 59, 64]. Patients often present with undefined lateral ankle pain or lateral ankle giving way complaints and typically demonstrate recognizable pain on palpation located over the posterior part of the distal fibula, worsened by activity [64].

Injury of the peroneal tendons can be debilitating for patients. Prompt diagnosis is the first step in the pathway of treating peroneal tendon tears. Depending on the severity of the pathology, different surgical treatment options are proposed [25, 46]. When less then 50 % of the cross-sectional tendon is involved, tears are often treated with debridement and tubularization of the tendon. Involvement of more then 50 % of the cross-sectional tissue may necessitate tenodesis to the adjacent intact peroneal tendon when it remains functional, or grafting when both tendons are found to be non-functional [25, 46, 64]. In the case of an acute complete rupture, both ends may be sutured together, although in chronic cases some form of tenodesis or tendon interposition is required to restore peroneal integrity. In symptomatic patients, surgical treatment has been associated with improved return to full activity and improvement in patients-reported outcome scores [13].

To optimize recovery of surgically treated peroneal tendon tears and ruptures, an appropriate rehabilitation programme is necessary. Facilitation of early return to activity is of great importance, since peroneal tendon tears are mostly found in active patients and athletes. Both non-weight-bearing immobilization (NWB) and weight-bearing immobilization (WB) are used in the rehabilitation process to facilitate an optimal recovery while preventing re-injuries. Since flexor tendons tend to form adhesions between the repaired tissue and surrounding scar tissue after surgical repair, early range of motion (ROM) is recommended in several tendon pathologies [18]. No evidence can be found, however, as to specifically when to begin ROM exercises following surgical repair of peroneal tendon tears and ruptures.

The aim of this study is to create an overview of available best practice evidence in the current literature with respect to rehabilitation options following surgical treatment of peroneal tendon tears and ruptures.

## Materials and methods

### Search strategy

Searching PubMed/MEDLINE and EMBASE electronic databases identified relevant literature. Three keywords (peroneal, tendon and tear) and related synonyms were used. All synonyms were combined with the Boolean command AND and were linked by the Boolean command OR. The last search was performed on 25 June 2015.

### Eligibility criteria

Original studies were included if (1) the study reported on peroneal tendon tears or ruptures, (2) the rehabilitation process after surgical treatment was described, (3) duration of immobilization was described, (4) the study was published after December 1994, and (5) full text was available in English.

### Study selection

Two authors (PAD, BL) performed the literature search and independently reviewed the search results. Titles and abstracts were reviewed by applying strict inclusion criteria for study characteristics as described above. Consensus for studies to be included was achieved by discussion between the two reviewers based on the predetermined selection criteria. Identified articles were reviewed on full text, and each reference list was screened for additional citation tracking.

### Data extraction

All data items were predetermined and specified as shown in Table 1. Two authors performed data extraction independently, using a modified extraction form. Duration of immobilization was described and rounded in weeks.

**Table 1:** Baseline characteristics

Study	Study design	Treatment	NWB in weeks	WB (partial) in weeks	Start ROM (weeks)
Arbab et al. [1]	II	A, B, D	2	4	6
Bare et al. [2]	II	A, B	4	4	5
Berg et al. [4]	III	D	2	6	–
Blitz and Nemes [5]	III	B	8	2	2
Bonnin et al. [6]	II	A	0	6, 5	–
Borland et al. [7]	III	A	6	0	6
Borton et al. [8]	III	C	6	0	6
Cerrato et al. [10]	I	B, C	2	4–6	2
De Yoe et al. [12]	III	A	4	2	4
Demetracopoulos et al. [13]	II	A	2	2	4
Dombek et al. [14]	II	A, B	4–5	2–3	4
Fujioka et al. [16]	III	B	2	2	2
Ho. et al. [19]	III	A	6	6	6
Jockel et al. [21]	II	C	8	4	12
Karlsson et al. [24]	II	A	0	6	6
Karlsson and Wiger [23]	I	A	0	6	2
Krause and Brodsky [25]	II	A, B	5	1–8	5
Lagoutaris et al. [26]	III	A	4	0	4
Lucas et al. [27]	III	A	2	4	6
Lui et al. [28]	I	A	4	0	–
Maurer and Lehrman [30]	III	B	6	3	5
	III	B	6	2	2
Madsen et al. [29]	III	C + D	8	0	–
Minoyama et al. [31]	III	A	2	2	–
Mook et al. [34]	II	C	4	2	3
Ochoa et al. [36]	III	A	4	0	4
Ousema and Nunley [37]	II	C	2	10	6
Ozer et al. [38]	III	C	4	0	4
Palmanovich et al. [39]	III	A	0	0	3
Patterson et al. [40]	III	B	1	5	6
Pelet et al. [41]	III	D	6	4	5
Pelligrini et al. [42]	III	C	2 + 4	0	5
Philbin et al. [43]	I	A, B, C, D	10 days + 4–6 weeks	4–6	8
Radice et al. [44]	III	B	5	0	5
Rapley et al. [45]	II	C	1	5	1
Redfern and Myerson [46]	II	B,C	6	6–8 (max. 3 months)	0
Ritter et al. [47]	I	A, B, C, D	2 + 2	2	2–4
Ross et al. [48]	III	A	2	3	–
Sammarco [49]	I	A, B	2	2	3
		C	4	2–3	6–7
Sammarco [50]	II	A	3	0	3
		C	4	2	
Saxena and Pham [53]	II	A	2–3	2	2
Saxena and Wolf [54]^a^	II	B	3	3	6
Shoda et al. [56]	III	A	6	0	–
Squires et al. [61]	I	A, B, C	6	4	3
Stockton et al. [62]	II	A, B	4	4 + 4	12
Vega et al. [65]	I	A	2	0	2
Verheyen et al. [66]	III	D	2	6	–
Waldecker et al. [67]	III	B	6	0	6
Wapner et al. [69]	II	A, B, C	0	3	3

Study design: *I* review/descriptive paper, *II* case series, *III* case report, treatment: *A* debridement with or without suturing, *B* tenodesis, *C* grafting, *D* end-to-end

^a^Reported on both technique A and B. Since results from technique A are already reported in an prior study from the same author, only the results from technique B are included

### Statistical analysis

Descriptive statistics including means and standard deviations were calculated for each variable. One-way ANOVA was used for the comparison of group means in duration of immobilization and time of start with ROM exercises, and post hoc analyses using Bonferroni correction were employed. A *p* value of less than 0.083 (0.05 divided by 6) was considered as statistically significant. Statistical analysis was performed using Stata (version 13.0, STATA Corp., TX, USA).

## Results

**Fig. 1:**
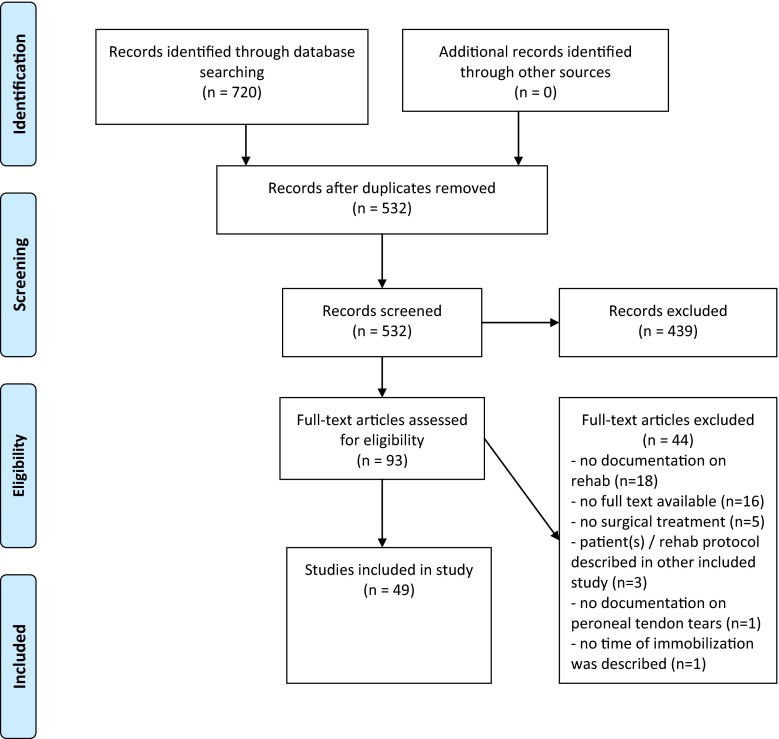
PRISMA flow diagram [33]

The literature search in PubMed/MEDLINE and EMBASE databases yielded, respectively, 421 and 299 records. After duplicates were removed, 532 studies were included for title and abstract review. Careful systematic selection resulted in 49 studies eligible for this review; 24 case reports, 8 reviews, and 17 case series (Fig. 1). No studies were found with the primary focus on rehabilitation of surgically treated peroneal tendon tears or ruptures. Included studies described their rehabilitation method after one or more of the following surgical treatment methods: group A: primary repair with or without tubularization of the tendon, [1, 2, 6, 7, 12–14, 19, 23–28, 31, 36, 39, 43, 47–50, 53, 56, 61, 62, 65, 69] group B: tenodesis, [1, 2, 4, 5, 10, 14, 16, 25, 30, 40, 43, 44, 46, 47, 49, 50, 61, 62, 67, 69] group C: grafting [8, 10, 21, 29, 34, 37, 38, 42, 43, 45, 46, 49, 50, 61, 69] and group D: end-to-end suturing [1, 29, 41, 43, 47, 66, 68]. Fourteen studies reported two or more surgical treatment methods [10, 25, 29, 43, 46, 47, 49, 50, 61, 62, 69]. Study characteristics and rehabilitation protocols are shown in Table 1.

### Rehabilitation after primary repair

Twenty-eight studies reported on the rehabilitation protocol after primary repair of the peroneal tendons. Some of these also included performance of side-to-side suturing or tubularization [1, 2, 6, 7, 12–14, 19, 23–28, 31, 36, 39, 43, 47–50, 53, 56, 61, 62, 65, 69]. The median duration of the immobilization period was 6.0 weeks (range 0–12) (Table 2). Of the studies (*n* = 23) that reported on the start of ROM exercises, 9 studies (39 %) started exercises within 4 weeks post-operative [23, 39, 47, 49, 50, 53, 61, 65, 69].

**Table 2:** Overview of the non-weight-bearing and weight-bearing immobilization period and the moment of start with Range of Motion per treatment group

	Group A: primary repair (*n* = 28)	Group B: tenodesis (*n* = 21)	Group C: grafting (*n* = 16)	Group D: end-to-end suturing (*n* = 7)
Total immobilization in weeks	Median 6.0 (range 0–12)	Median 7.0 (range 3.0–13)	Median 6.3 (range 3.0–13)	Median 8.0 (range 6.0–11)
NWB in weeks	Median 3.5 (range 0–6.4)	Median 4.3 (range 0–8.0)	Median 4.0 (range 0–8.0)	Median 4.0 (range 2.0–8.0)
WB in weeks	Median 2.3 (range 0–8.0)	Median 3.0 (range 0–8.0)	Median 2.8 (range 0–10)	Median 4.0 (range 0–6.0)
Start ROM in weeks	*n* = 23^a^	*n* = 20^a^	*n* = 15^a^	*n* = 4^a^
	Median: 4.0 (range 2.0–12)	Median: 4.5 (range 0–12)	Median: 4.0 (range 0–12)	Median: 5.5 (range 2.0–8.0)

*NWB* non-weight bearing, *WB* weight bearing, *ROM* range of motion

^a^Number of studies that reported on the start of range of motion after surgery

### Rehabilitation after tenodesis

Rehabilitation after tenodesis was reported in 21 studies [1, 2, 4, 5, 10, 14, 16, 25, 30, 40, 43, 44, 46, 47, 49, 50, 54, 61, 62, 67, 69]. The median duration of immobilization was 7.0 weeks (range 3.0–13) (Table 2). Of the studies (*n* = 20) that reported on the start of ROM exercises, 9 studies (45 %) started exercises within 4 weeks post-operative [5, 10, 16, 30, 46, 47, 49, 61, 69].

### Rehabilitation after grafting

Rehabilitation after surgical treatment with grafting was reported in 16 studies [8, 10, 21, 29, 34, 37, 38, 42, 43, 45, 46, 47, 49, 50, 61, 69] with a median immobilization period of 6.3 (range 3.0–13) weeks (Table 2). Of the studies (*n* = 15) that reported on the start of ROM, 7 studies (47 %) reported on a start within 4 weeks post-operative [10, 34, 45–47, 61, 69].

### Rehabilitation after end-to-end suturing

Seven studies [1, 29, 41, 43, 47, 66, 68] described the rehabilitation method after tendon end-to-end suturing technique. The median immobilization period was 8.0 weeks (range 6.0–11) (Table 2). Of the studies (*n* = 4) reporting on the start of ROM, 1 study (25 %) started exercises within 4 weeks post-operative [47].

### Comparison of groups

There was no difference with respect to the total duration of immobilization between the different treatment groups (*n.s.*). Furthermore, when NWB and WB duration rates among different treatment groups were compared, no difference was found (*n.s.*).

## Discussion

The most important finding of this study was that there exists a wide variation in rehabilitation protocols provided after surgical treatment of peroneal tendon tears and ruptures, confirming that there is no consensus among foot and ankle providers. No difference could be found in post-operative protocols between different treatment options. In recent years, there seems to be a trend towards early ROM and rehabilitation within 4 weeks post-operative [13, 23, 24]. However, it is difficult to formulate conclusions based on these data since current literature lacks studies that were primarily designed to study specific rehabilitation methods.

Peroneal tendon injuries are common in active patients. For this patient population, early return to activity and sports is of great importance. Since peroneal tendon tears and ruptures are protracted injuries, surgical repair merely marks the beginning of a long recovery period. Adequate rehabilitation is purported as an important aspect of the clinical success of any operatively treated tendon injury. A properly directed rehab programme can facilitate tendon healing, minimize scarring and promote early return to pre-injury activity/sports levels. Great attention should therefore be paid to determining the optimal post-operative treatment protocol.

Many rehabilitation recommendations have been published over the past decade regarding flexor tendons of the hand [18]. Flexor tendons are predisposed to forming adhesions between the repair and surrounding tissue, leading to scar, loss of ROM and limitation of tendon gliding. To prevent adhesion formation, early ROM is recommended [3, 11, 15, 52]. Different authors have also advocated early post-operative rehabilitation after Achilles tendon surgery [20, 22, 35, 60, 63]. A recent change to early ROM exercises can be found in operatively treated patients with peroneal tendon injuries [13, 23]. Demetracouplos et al. and Karlsson et al. [13, 23, 24] have recently described a change in their post-operative management based on this information. In contrast to a previous protocol of 6 weeks cast immobilization followed by physical therapy, Demetracouplos et al. [13] implemented a post-operative protocol aiming early ROM after 4 weeks of WB and NWB immobilization. Karlsson et al. [23, 24] immobilized the patient 6 weeks in a plaster cast, but shortened the period in a study published 4 years later to 2 weeks plaster cast followed by a WB air cast brace to provide early ROM training.

Among the available studies analysed, we found wide variation in the total immobilization period. While some authors preferred early ROM without post-operative immobilization, [39] others immobilized their patients over 12 weeks [46]. Due to the wide range found for this period of inactivity (0–13 weeks) among different studies, it is hard to draw conclusions and propose an evidence-based rehabilitation protocol. Based on our own experience, we recommend that an ideal peroneal rehabilitation protocol should be tailored according to individual patients needs and should be dependent upon the exact nature of tendon injury as well as the functional expectations of each patient.

This study has a few limitations. First, the clinical heterogeneity and small patients numbers among the included studies withholds us from drawing hard conclusions and therefore establishing an evidence-based protocol. Secondary, the results of this study were based on reviews and studies with the primary focus on the operative treatment of these ruptures. These methodological limitations prevented high-quality conclusions based on synthesis of the available evidence. Therefore, our results provide an overview on the daily affairs in clinic and do not provide a sufficiently evidence-based recommendation and thus no statement can be made on the effectiveness of the rehabilitation protocols currently being employed. Our analysis, however, is based on best available evidence suggesting broad variation between different surgeons and lack of any consensus on a post-operative peroneal protocol. Finally, the search we performed yielded a relatively large amount of unavailable manuscripts.

### Proposed rehabilitation programme

In order to come up with an evidence-based algorithm for the rehabilitation of peroneal disorders in daily clinical practice, a programme is proposed based on evaluation of available protocols described in today’s literature as well as personal experience of the centres involved in this study. It is emphasized that this protocol will ultimately require validation.

**Fig. 2:**
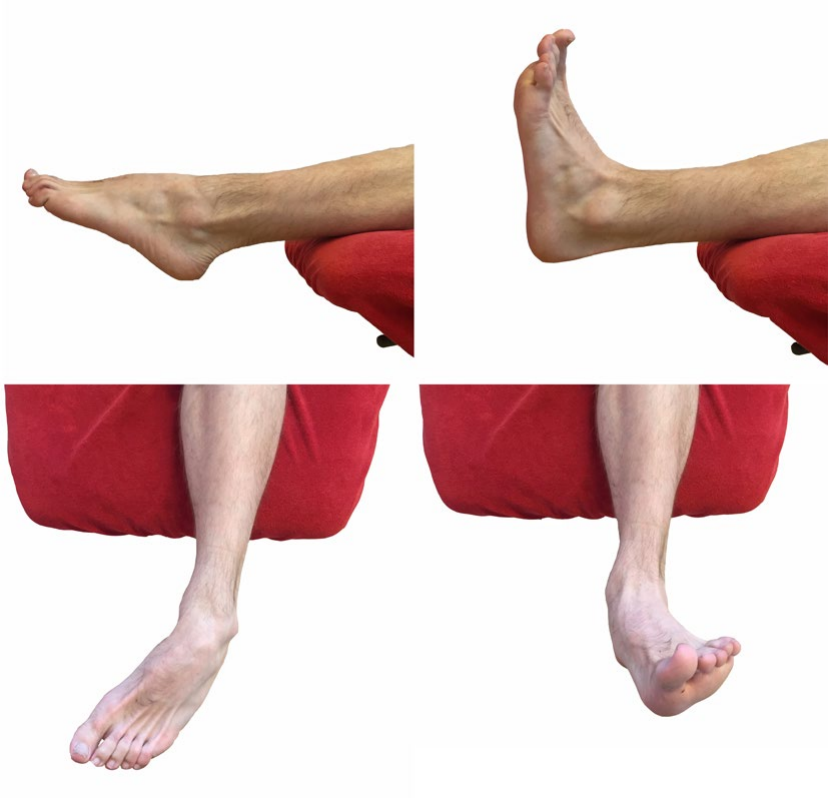
Patient can start with active full ROM exercises: dorsiflexion, plantar flexion, inversion, eversion

**Fig. 3:**
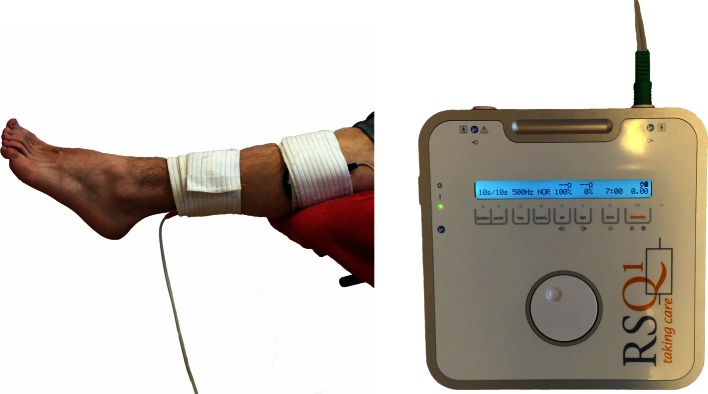
Strength exercises: using the RSQ1 for electrical stimulation. In the second phase you can use this device during isometric or isotonic exercises

**Fig. 4:**
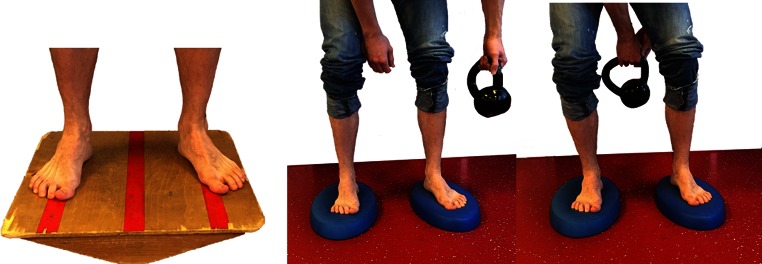
Proprioceptive training: progress from NWB/controlled WB on two legs to full WB on unstable surfaces

**Fig. 5:**
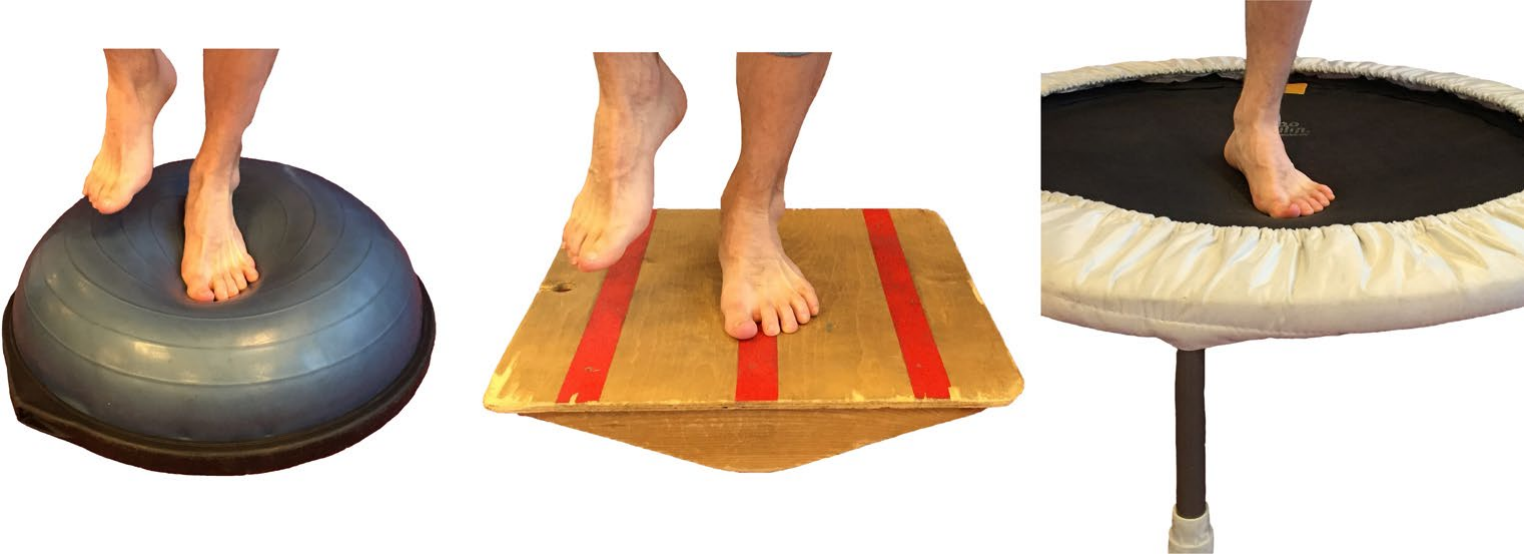
Single leg balance activities (stable to unstable surfaces, without and with distractions)

**Fig. 6:**
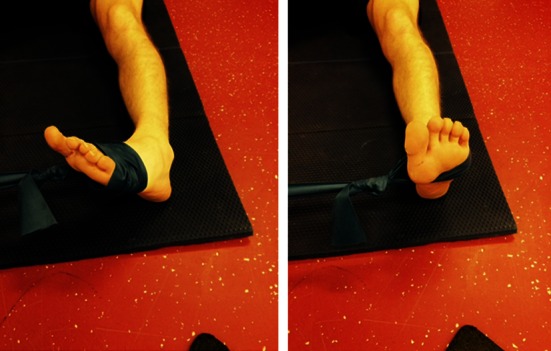
Strength exercises: eversion against theraband. This is one of the most important exercises

**Fig. 7:**
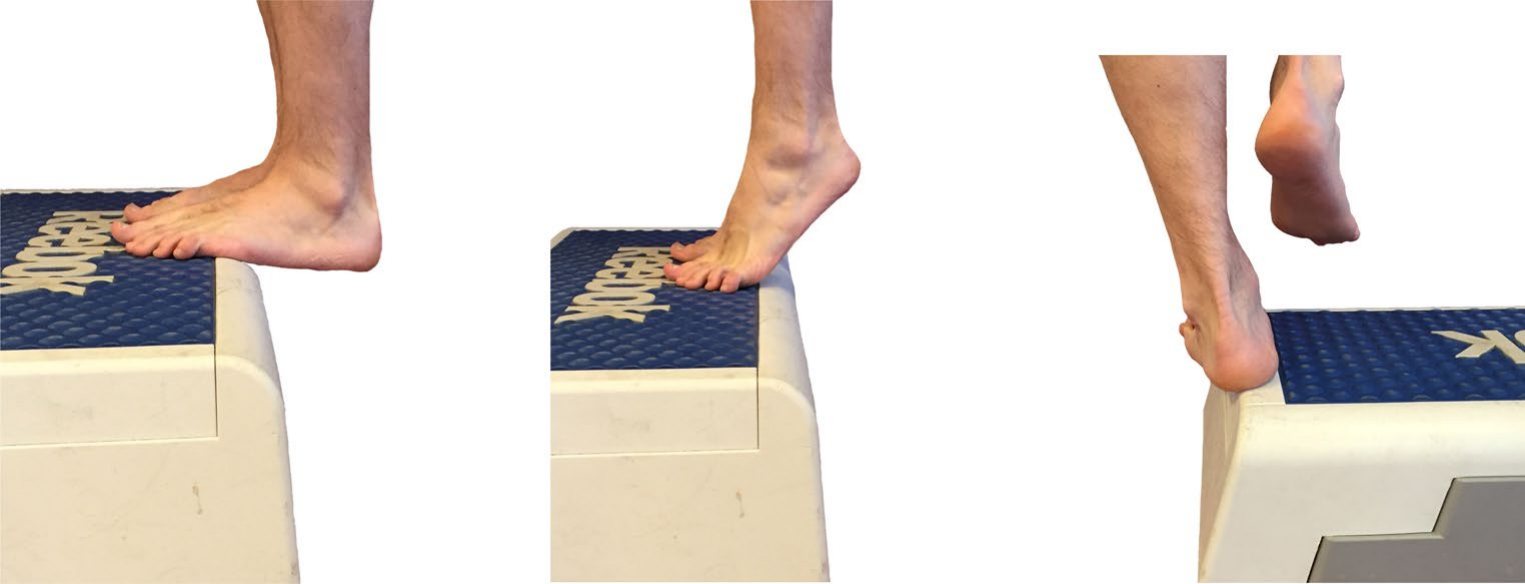
Training of the strength of the foot and calf muscles

**Fig. 8:**
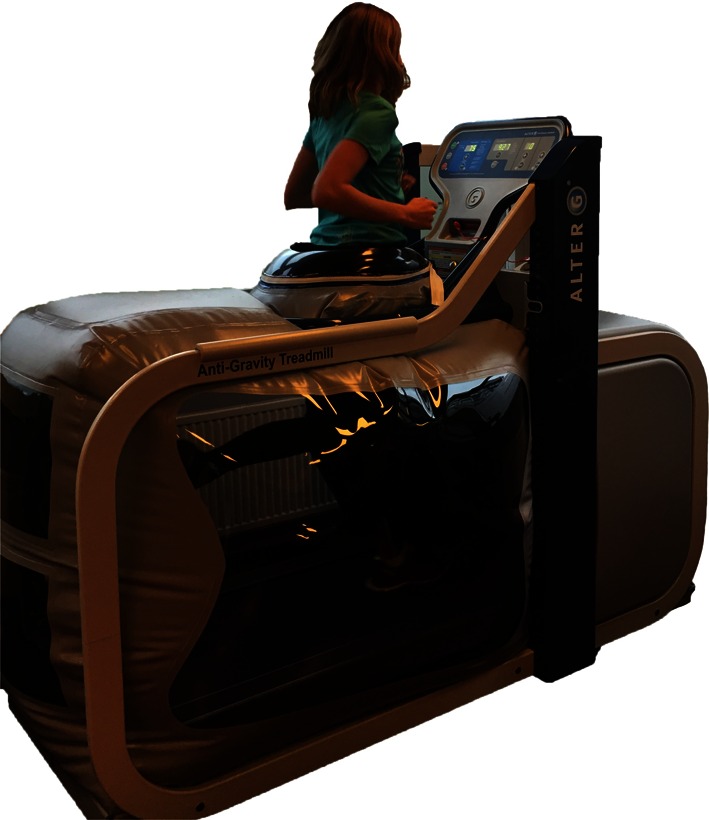
Walking in an Alter-G trainer

Following surgical treatment of peroneal tendon tears, patients should receive a post-operative lower leg splint for 2 days, followed by 12 days of a NWB lower leg cast. After removal of the stitches, patients are then permitted to weight bear in a walker boot or lower leg cast for 4 weeks pending surgeon preference. Six weeks post-operative, physiotherapy is initiated to restore ROM (Fig. 2) and strength. Strength exercises include isometric exercises in pain free range and electrical stimulation of the peroneal muscles (Fig. 3). Simultaneously, proprioception and balance are trained by seated or partial WB exercises and proprioceptive exercises on two legs (Fig. 4). Proprioceptive exercises are gradually expanded from controlled WB on two legs to full WB on two legs (Fig. 5). Eccentric, concentric and isotonic exercises are also started with the use of a theraband (Fig. 6). The strength of the foot and calf muscles is trained (Fig. 7), and the walking pattern is checked. Patients start to learn to walk again in a controlled setting either with use of an Alter-G trainer (Fig. 8) or a swimming pool in order to allow good motion in a partial WB setting to start with. This is helpful in preventing development of reactive peroneal tendinitis. No provocation of the peroneal tendons is allowed until 12 weeks post-operatively, and sports-specific rehabilitation is generally not initiated until at least 12 weeks of physiotherapy have concluded.

An overview of the proposed rehabilitation protocol is shown in Table 3. It is important to emphasize that the number of weeks are a median number of weeks and that each rehabilitation programme should be tailored according to individual patient needs, depending on both the exact nature of the peroneal problem as well as on the specific personal demands of the specific patient.

**Table 3:** Overview of the proposed rehabilitation protocol of surgically treated peroneal tendon disorders, based on the evaluation of available protocols in literature

	0–2 weeks^a^	2–4 weeks^a^	6–8 weeks^a^	8–12 weeks^a^	12–24 weeks^a^	>24 weeks^a^
Weight bearing:						
1. Non-weight bearing	x					
2. Partial weight bearing		x	x			
3. Full weight bearing				x	x	x
Active Range of Motion			x			
Strength exercises			x			
Proprioceptive training			x	x		
Eccentric/concentric exercises				x	x	
Isotonic exercises				x	x	
Running					x	x
Sport specific training						x
Provocation peroneal tendons						x

^a^Number of weeks after operation

## Conclusion

Rehabilitation is an important factor in the clinical success of all tendon injuries, and treatment of peroneal tendon tears and ruptures is no exception. There is no consensus in today’s literature with regard to an ideal post-operative immobilization time or initiation of range of motion exercises. Prospective, randomized controlled trials are needed to refine optimal rehabilitation methods for patients with peroneal tendon tears or ruptures after operative treatment. Based on currently available data and a combined personal clinical experience exceeding 50 years, a tailored rehabilitation protocol for every specific patient is advised for optimal functional recovery and prevention of re-rupture.
